# Laser-Induced Breakdown Spectroscopy Combined with Nonlinear Manifold Learning for Improvement Aluminum Alloy Classification Accuracy

**DOI:** 10.3390/s22093129

**Published:** 2022-04-20

**Authors:** Edward Harefa, Weidong Zhou

**Affiliations:** Key Laboratory of Optical Information Detection and Display Technology of Zhejiang, Zhejiang Normal University, Jinhua 321004, China; edwardharefa@zjnu.edu.cn

**Keywords:** LIBS, dimensionality reduction, manifold learning, classification, Isomap, Laplacian eigenmaps, local linear embedding, local tangent space alignment

## Abstract

Laser-induced breakdown spectroscopy (LIBS) spectra often include many intensity lines, and obtaining meaningful information from the input dataset and condensing the dimensions of the original data has become a significant challenge in LIBS applications. This study was conducted to classify five different types of aluminum alloys rapidly and noninvasively, utilizing the manifold dimensionality reduction technique and a support vector machine (SVM) classifier model integrated with LIBS technology. The augmented partial residual plot was used to determine the nonlinearity of the LIBS spectra dataset. To circumvent the curse of dimensionality, nonlinear manifold learning techniques, such as local tangent space alignment (LTSA), local linear embedding (LLE), isometric mapping (Isomap), and Laplacian eigenmaps (LE) were used. The performance of linear techniques, such as principal component analysis (PCA) and multidimensional scaling (MDS), was also investigated compared to nonlinear techniques. The reduced dimensions of the dataset were assigned as input datasets in the SVM classifier. The prediction labels indicated that the Isomap-SVM model had the best classification performance with the classification accuracy, the number of dimensions and the number of nearest neighbors being 96.67%, 11, and 18, respectively. These findings demonstrate that the combination of nonlinear manifold learning and multivariate analysis has the potential to classify the samples based on LIBS with reasonable accuracy.

## 1. Introduction

Aluminum alloys are one of the most widely utilized nonferrous metal structural elements in the industry [1]. Thus, regardless of the stage of production and manufacture, or the process of detection and recycling, it is critical to categorize aluminum alloys quickly and adequately by employing a reliable analytical method, as this has significant practical implications and value. Conventional chemical analysis of the elemental composition, such as X-ray fluorescence (XRF), atomic absorption spectrometry (AAS), and inductively coupled plasma-atomic emission spectrometry (ICP-AES) [2,3,4], have been used as methods for identification of content in soils. However, these detection methods are extremely time-consuming, expensive, and require rigorous sample preparation, which makes them incompatible with real-time detection and eco-friendly analysis.

We propose laser-induced breakdown spectroscopy (LIBS) as an analytical method to classify aluminum alloys because the LIBS enables the quick acquisition of valuable spectroscopic data from a wide type of materials (e.g., solids, liquids, or gases) without complex sample preparation, with fast detection, while remaining less disruptive, and inexpensive [5,6,7]. An intensive laser beam is utilized in LIBS to create breakdown, i.e., a plasma, on the surface of a sample, resulting in simultaneous atomization and excitation. Plasma light carries information about the elemental composition in the sample. Nowadays, LIBS has been implemented in a wide range of applications, such as industrial application [8], underwater detection [9], food analysis [10], cultural heritage [11], environmental monitoring [12], space exploration [13], medical diagnosis [14], and many other fields. The effectiveness of the classification results is determined not only by the training set data process but also by the sophistication and competence of the methodologies used to classify data from unknowns. In recent years, advances in multivariate analysis as classifiers have aided in interpreting datasets from diverse LIBS applications. For example, when using a random forest (RF) algorithm as a classifier for iron ore classification, Sheng et al. obtained an accuracy rate for the training test and the test set and were 98.50% and 96.00%, respectively [15]. Zhao et al. employed a support vector machine (SVM) to discriminate the geographical origins of all honey, multi-floral honey, and acacia honey and found that SVM performed satisfactory results [16]. Lee et al. aimed to classify edible salts from 12 various geographical origins, and they proposed soft independent modeling of class analogy (SIMCA) as a classifier method. They achieved a 97% classification accuracy in the test dataset using the SIMCA method [17].

On the other hand, Xu et al. [18], Weng et al. [19], and Boucher et al. [20] highlighted that using multivariate analysis directly in the high-dimensional dataset would not be practical and reliable for analysis. In fact, because of the complexity of aluminum’s elemental composition and the advancement of the spectrometer, the acquired LIBS spectra often comprise numerous emission lines of varying intensity. Consequently, retrieving trustworthy information from raw spectra data and lowering the original data dimensions have been immensely demanding in LIBS applications [21,22,23]. Dimensionality reduction is a challenging process and yet a fundamental task in many pattern recognition problems and machine learning applications. Numerous advanced techniques in dimensionality reduction exist, and each is based on a different set of assumptions and conditions. These techniques can be generically classified as linear or nonlinear. The most frequently linear techniques that have been employed in LIBS analysis are linear discriminant analysis (LDA) and principal component analysis (PCA) [24,25,26]. Migenda et al. and Kemfert et al. emphasized that when the input dataset is completely linearly connected, linear approaches can adequately learn a linear structure. However, when data is highly nonlinear in structure, the conventional linear technique can fail to present and demonstrate the true structure of the dataset [27,28].

To address the issue, we propose nonlinear manifold learning as dimensionality reduction techniques, such as Laplacian eigenmaps (LE), local tangent space alignment (LTSA), local linear embedding (LLE), and isometric mapping (Isomap) [29]. Isomap is a global method for generating a low-dimensional embedding while retaining the pairwise distances between data points. LLE is a technique that generates low-dimensional embeddings of high-dimensional data while keeping their locality. It makes use of the linear reconstruction’s local symmetries to uncover nonlinear structures in high-dimensional data. Compared to LLE, LTSA constructs the embedding by utilizing the tangent space of each data point and aligning those local tangent spaces. LE is a manifold learning algorithm that utilizes the manifold’s local attributes to generate a low-dimensional dataset [30,31]. With the uniqueness and benefit of the nonlinear manifold learning algorithm, this study investigates and compares the performance of nonlinear and linear manifold dimensional reduction techniques for reducing the dimension in high-dimensional spectral data for improving aluminum alloy classification accuracy. Furthermore, there has been no exploration of the implementation of nonlinear manifold learning in LIBS applications.

## 2. Materials and Methods

### 2.1. Experimental Work

**Figure 1:**
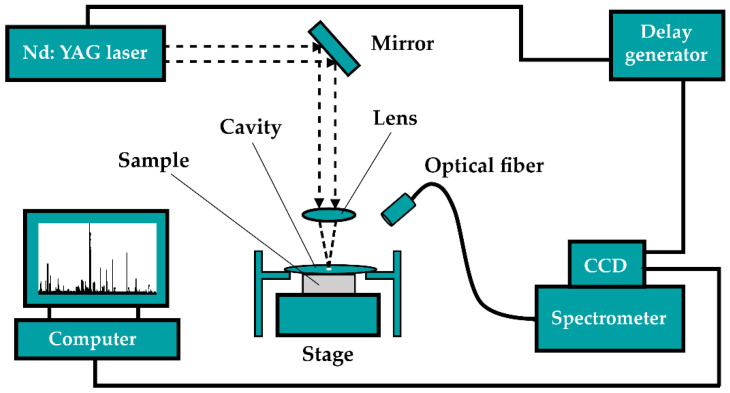
The LIBS experimental setup.

The schematic of the LIBS experimental device is illustrated in Figure 1. The setup consisted of a laser source, optical system, fiber spectrometer, digital pulse delay generator, and data acquisition computer. The working wavelength of the Q-switched Nd: YAG laser (Vlite-200, Beamtech, China) was 1064 nm, the pulse width was 10 ns, the pulse energy was 30 mJ, and the laser working frequency was set to 1 Hz. In the experiment, the focused laser spot on the target surface was measured to be 450 μm in diameter, resulting in a laser fluence of 18.9 J/cm^2^ and an irradiance of 1.89 GW/cm^2^ delivered to the sample. The laser beam was reflected through the mirrors and then focused using the convex lens with a focal length of 70 mm on the sample surface to generate laser plasma. Moreover, a cylindrical cavity with a height of 1 mm and 3 mm diameter was placed on the target surface to optimize the signal-to-noise ratio and emission intensity. The experimental sample was placed on a two-dimensional movable platform. The digital pulse delay generator (DG535, Standford Research System, USA) was used to control the time delay between the laser pulse and the external trigger of the spectrometer. The collimating lens was placed 2 mm away at a 30° angle from the laser beam to capture the plasma emission, and then a bundle of optical fiber with a diameter of 200 μm delivered the collected light to a multi-channel fiber optic spectrometer (AvaSpec-ULS2048-USB2, Avantes, The Netherlands). The spectrometer had an average resolution of 0.08 nm and was equipped with a linear charge-coupled device (CCD) detector with 2048 pixels. The detector can be externally triggered to initiate spectroscopy recording with a delay time of 1 μs, gate width was fixed at 2 ms, and the measurement range was 190 nm to 510 nm.

The sample tested in the experiment was the BYG2163 6063 standard aluminum alloy set, which was purchased from the National Institute of Metrology, China. This set consisted of five cylinder blocks of aluminum alloy with a diameter of 50 mm and thickness of 30 mm for each block, and their chemical contents are presented in Table 1. For each experiment, 500 spectra were obtained throughout the spectra acquisition process. Ten spectra were averaged and utilized as one replication analysis spectrum, resulting in fifty independent replicate spectra for each experiment. The acquired spectra were stored in the computer for further analysis.

### 2.2. Nonlinearity Test and Nonlinear Manifold Learning Algorithms

Before performing nonlinear manifold learning on the dataset, we confirmed the existence of nonlinearity conditions in the dataset by employing the augmented partial residual plot [32,33]. In this study, we explored and implemented the plot by correlating the first *n* principal components (*PC*s) of the predictor *X* and the square of the first *PC* with the response *Y*:

(1) $$y_{i}=b_{0}+b_{1}\cdot PC_{1}+\ldots +b_{n}\cdot PC_{n}+b_{mm}\cdot PC_{m}^{2}+e_{augpres}$$

where *m* = 1, 2, …, *n*, the coefficient of respective of *PC* is symbolized as *b*, and *e_augpres_* defines the fitting residual. The detection of the nonlinearity figure was achieved by plotting the sum $e_{i}=e_{augpres}+b_{m}\cdot PC_{m}+b_{mm}\cdot PC_{m}^{2}$ against the *PC_m_*.

The local tangent space alignment (LTSA) is a nonlinear manifold dimensionality reduction technique that seeks to discover a system of global coordinates inside the low-dimensional space that adequately represents the high-dimensional dataset [29]. LTSA calculates the *k* nearest neighbors from each data point $x_{i}$, i ∈ M, and constructs a centralized matrix of neighbors *M_i_* which also includes $x_{i}$. Following that, it closely resembles the *d*-dimensional tangent space $\Theta_{i}$ of every neighborhood by determining the first *d* right singular vectors of $M_{i}$ according to the respective *d* biggest singular values. The effectiveness of LTSA is heavily dependent on the accuracy of the estimation of the local tangent spaces, which implies that if the datasets are not precisely located on *d*-dimensional coordinates, this estimate will be quite severe. Thus, prior to applying the LSTA technique to obtain the intrinsic spectral variables, the original spectrum dataset is pretreated to remove noise and improve the approximate smoothing manifold surface construction [34,35]. 

To maintain the local geometry of the input information in low-dimensional space, locally linear embedding (LLE) seeks to reconstruct the global topology of nonlinear manifolds using locally linear approximations. LLE depicts each point $x_{i}$ as a linear mixture of its neighbors once the graph of the neighborhood is formed using the Euclidean distance.

(2) $$x_{i}=\sum_{j\in K_{i}}w_{ij}x_{j},\;i\in M$$

where $K_{i}$ defines the collection of parameters of the *k* nearest neighbors of $x_{i}$, and the generic weight $w_{ij}$ emphasizes the involvement of neighbor *j* for reconstructing point *i*. By optimizing the function, the weight coefficients for input datasets can be calculated as:

(3) $$\sum_{i\in M}\|x_{i}-\;\sum_{j\in K_{i}}w_{ij}x_{j}\;\|{}^{2}$$

which is constrained by the conditions $\underset{j\in K_{i}}{\sum }w_{ij}=1,\;i\;\in \;M$, implying that the weights are insensitive to rotations, rescales, and translations of associated neighbors and particular points.

Isomap is a nonlinear manifold-based modification of the multidimensional scaling (MDS) technique. In contrast to standard MDS, which seeks to maintain the Euclidean distance between data points, Isomap seeks an embedding in which the geodesic distance between two points in the input space is as near to the Euclidean distance between respective representations in the targeted space as possible [36,37]. Let $D_{G}$ indicate the matrix containing the distances of geodesic between the neighbors’ points. The embedding into the *d*-dimensional space is determined by optimizing the following operation:

(4) $${\left\|\tau \left(D_{G}\right)-\tau \left(D_{Z}\right)\right\|}_{F}$$

where the τ operator transforms the distances to the inner products, the matrix of pairwise Euclidean distances $d_{ij}=\|Z_{\;i}-Z_{j}\|$ of the data projections in ${ℜ}^{d}$ is symbolized as $D_{Z}=\left[d_{ij}\right]$, and ${\left\|•\right\|}_{F}$ represents the Frobenius norm of a matrix. The global minimum of Equation (4) obtained by determining the *d* eigenvectors correspond with the *d* biggest eigenvalues of the geodesic distances matrix $\tau \left(D_{G}\right)$.

To generate low-dimensional projections, LE uses the concept of the Laplacian of the neighborhood graph. The edge of the neighborhood graph, which linking point $x_{i}$ to one of the respective nearest neighbors $x_{j}$ is weighted by applying two different criteria: the projection $Z_{i},\;i\;\in \;M$ and the weight $w_{ij},\;i\;\in \;M,\;j\;\in {\;K}_{i}$ in the LE technique. The weight $w_{ij}$ values are determined using the Gaussian kernel function [38,39]:

(5) $$w_{ij}=\exp\left(-\frac{{\left\|x_{i}-x_{j}\right\|}^{2}}{t}\right),\;t\in ℜ$$

where *t* is the heat kernel parameter. Equation (5) assigns an increasing weight as the points $x_{i}$ and $x_{j}$ become closer together. The straightforward technique substitutes $w_{ij},\;i\;\in \;M,\;j\;\in {\;K}_{i}$. In both situations, $w_{ij}=0$ for $j\;\notin {\;K}_{i}$. Then, by mitigating the function, the projections $Z_{i},\;i\;\in \;M$ of the data points in the lower dimensional space are calculated:

(6) $${\sum_{i\in M,\;j\in K_{i}}\left\|z_{i}-z_{j}\right\|}^{2}w_{ij}$$

It imposes a severe penalty on neighboring points that are mapped at a considerable distance apart. The optimization of Equation (6) is reduced to the following minimization problem by incorporating the Laplacian matrix *L* = *D* − *W* of the neighborhood network, where *D* is a diagonal matrix with components $D_{ii},\;\underset{j\;\in \;M}{\sum }W_{ij},\;i\;\in \;M$.

(7) $$\min\;trace\left(Z^{\prime }LZ\right)$$

The simple form solution of Equation (7) is achieved by finding the *d* eigenvectors corresponding to the *d* lowest nonzero eigenvalues of the generalized eigenvalue problem Lv=λDv and setting the projections *Z* = *V*.

### 2.3. Multivariate Classifier and Evaluation Parameter

In this study, we randomly divided the LIBS data into 75% for the training set and 25% for the test set. Support vector machines (SVM) with a radial basis function (RBF) kernel were used as a multivariate analysis for samples classification in the comparison. SVM is a cutting-edge supervised learning algorithm that has been extremely successful in data classification and employed in the LIBS field to solve qualitative and quantitative analyses. There are two significant hyperparameters in the SVM model, namely the penalty parameter of the error term (*C*), and the gamma (*γ*) parameter that decides how much curvature we want in a decision boundary [16,37]. The grid search with ten-fold cross-validation was used to tune the hyperparameters and obtained the optimal values, for *C* was 10 and for *γ* was 0.1. 

We demonstrated the efficiency result of the method in this study using a confounding matrix to evaluate the accuracy of the classifier. It is a widely used and accepted approach and standard to evaluate classifier performance using this performance measure. The proportion of correctly assessed results in the total observed values in the classification model is used to determine the model’s overall discriminant classification ability. The following equation is used in the calculation [40]:

(8) $$\mathrm{Accuracy}=\frac{\mathrm{TP}+\mathrm{TN}}{\mathrm{TP}+\mathrm{TN}+\mathrm{FP}+\mathrm{FN}}$$

The output of a classification analysis has only two possible values: positive (P) or negative (N). Variable P, for example, related to aluminum alloy sample #1 in our case, while N correlated with other samples. For the binary classifier, there are four potential outcomes. If the forecasted output is sample #1 and the actual input is sample S#1 or other samples, a true positive (TP) or a false positive (FP) is detected. In contrast, if the forecasted output is other samples and the actual input is other samples or sample #1, respectively, a true negative (TN) or a false negative (FN) is observed.

## 3. Results and Discussion

**Figure 2:**
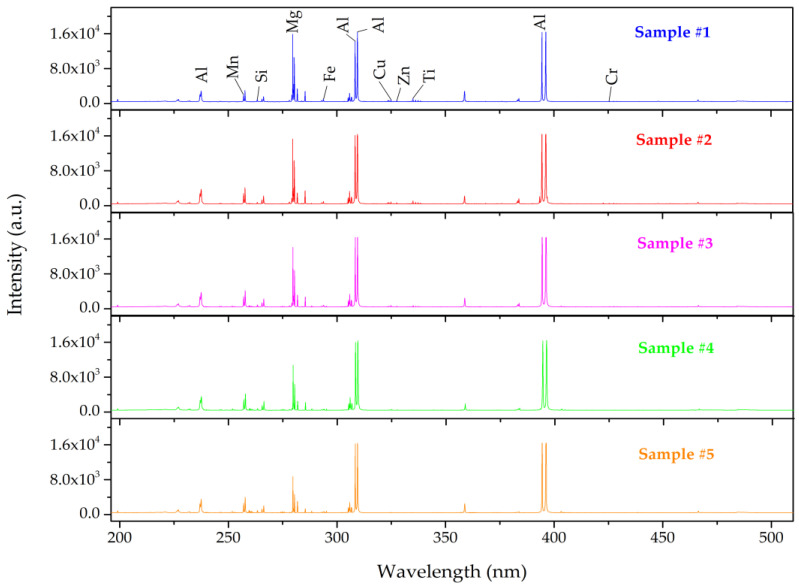
Average LIBS spectra of the five aluminum alloys.

The average acquired emission spectra of five aluminum alloy samples ranging from 190 nm to 510 nm are illustrated in Figure 2. We could identify the eight major elements in the samples and their associated spectral emission lines using the National Institute of Standard and Technology (NIST) atomic spectroscopy database [41], namely Al (237.31 nm, 256.80 nm, 257.54 nm, 265.35 nm, 281.62 nm, 308.22 nm, 309.27 nm, 394.40 nm, 396.15 nm), Si (198.63 nm, 263.12 nm), Fe (295.47 nm, 358.12 nm), Cu (324.75 nm), Mg (279.55 nm, 280.27 nm, 285.21 nm, 383.23 nm), Mn (257.09), Zn (328.23 nm), Ti (334.94 nm), and Cr (425.43 nm). It is clearly seen from Figure 2 that the spectra intensity of those alloys are so similar, and direct classification and identification are challenging to implement. Moreover, the raw LIBS dataset contains more than 4000 wavelengths in spectra and a number of intensities points for each sample; assigning all would have greatly increased the inaccuracy of predictive performance.

The classification accuracy using standard SVM on full spectra was 68.33%, and this result was not satisfactory. Therefore, we needed to implement dimensionality reduction methods.

**Figure 3:**
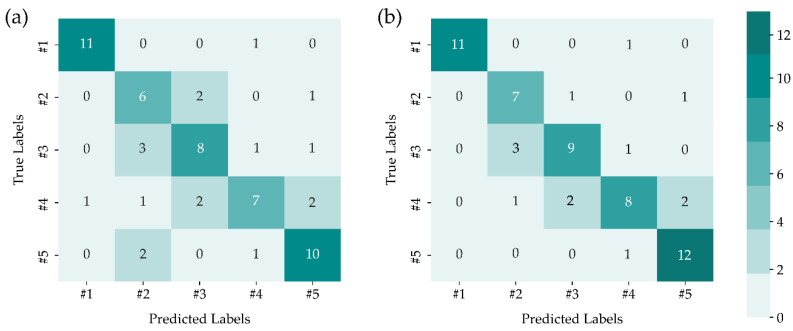
Confusion matrices for (**a**) PCA-SVM and (**b**) MDS-SVM models.

The preliminary exploration was conducted by combining SVM with linear manifold learning techniques, namely PCA and MDS, on LIBS data, and five-fold cross-validation was employed to determine the optimum number of dimensions (*n_dimensions_*). It can be seen from Figure 3 that only 1 data in sample #1 was misclassified as sample #4, both in PCA and MDS. Moreover, compared to that using PCA-SVM, the misclassified data in samples #2, #3, and #4 were decreased, and only 1 data in sample #5 was misclassified as sample #4 after implementing MDS-SVM. The highest classification accuracy of PCA-SVM was 70.00% with *n_dimensions_* = 23 and MDS-SVM was 78.33% with optimum *n_dimensions_* = 18, and these results need further improvement.

**Figure 4:**
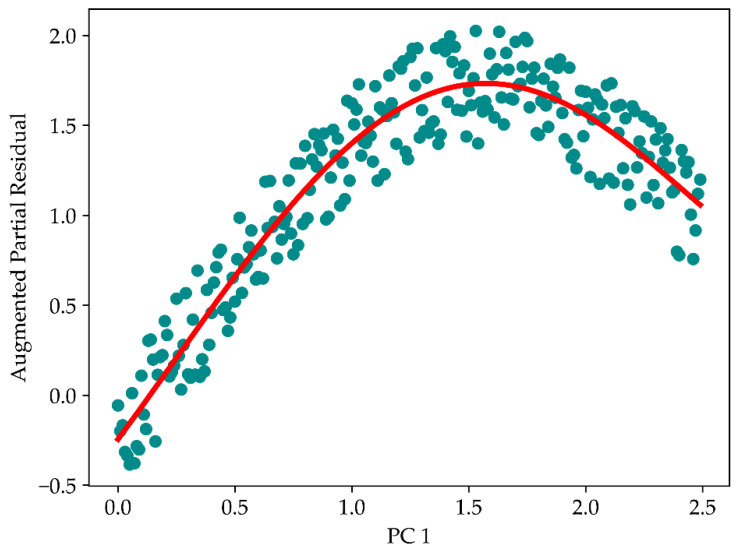
Determining nonlinearity in LIBS dataset using an augmented partial residual plot towards, where the first twelve s were included in the calculation and plotting.

The augmented partial residual plot method was employed to investigate whether there was nonlinearity in the LIBS data. The result of the polynomial fitting, shown in Figure 4, illustrated that there was a significant nonlinearity condition in the dataset.

**Figure 5:**
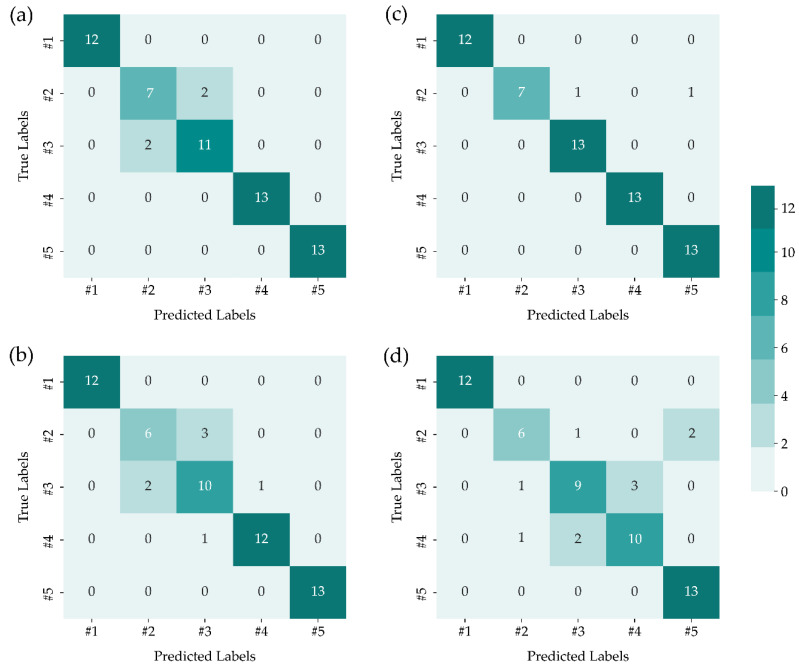
Confusion matrices of SVM prediction outcomes using various manifold dimensionality reduction techniques of (**a**) LTSA, (**b**) LLE, (**c**) Isomap, and (**d**) LE.

Four nonlinear manifold learning, i.e., LTSA, LLE, Isomap, and LE, were performed to address the nonlinear dimensionality reduction. The confusion matrix of the test set was presented to show the capability of the techniques. Each column in the confusion matrix represented occurrences belonging to a predicted label, whereas each row represented instances belonging to a true label. We first implemented LTSA-SVM in the test set, and the confusion matrix of LTSA-SVM is shown in Figure 5a. The LTSA-SVM successfully classified samples #1, #4, and #5, and only 2 data were misclassified in samples #2 and #3. Even though there is still inappropriate data classification, LTSA-SVM demonstrates better performance than PCA-SVM and PCA-MDS. The confusion matrix of the combination SVM and LLE is depicted in Figure 5b, and it was illustrated that all data in samples #1 and #5 were correctly classified with others, while 3 data of sample #2 remained misclassified as sample #3. When implementing LLE-SVM in samples #3 and #4, there were 3 and 1 data misclassified, respectively, and this result showed a reduction in performance of LLE-SVM compared to LTSA-SVM. Figure 5d exhibits the confusion matrix of the LE-SVM method, and this result showed that the LE-SVM could make the perfect distinction between samples #1 and #5 with other types. On the other hand, the LE-SVM method could not well separate samples #3 and #4 from sample #2, and some of the data in samples #2 and #4 were also misclassified as sample #3. As illustrated in Figure 5c, only sample #2 was misclassified as samples #3 and #5, and other samples were perfectly distinguished. The Isomap-SVM outperforms the data classification result compared to the other three nonlinear manifold learning.

**Figure 6:**
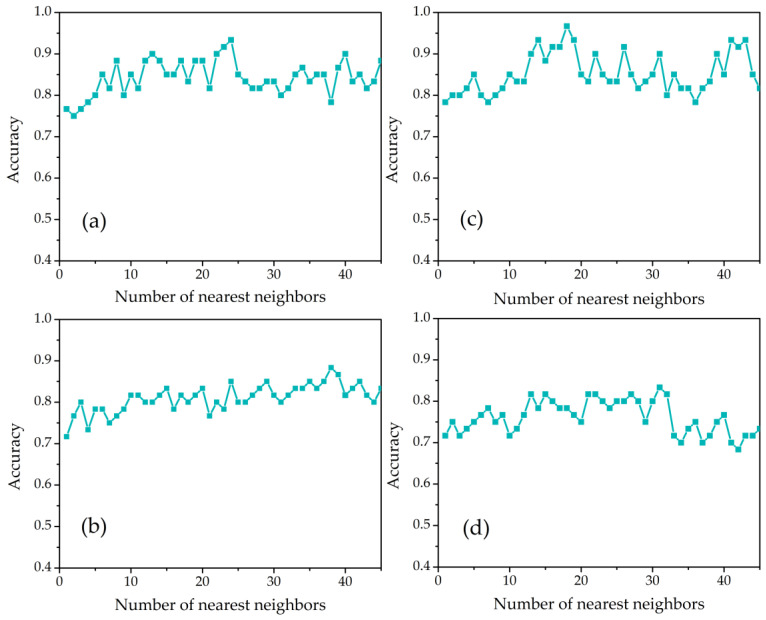
Classification accuracies of the number of nearest neighbors parameter variation in SVM combined with (**a**) LTSA, (**b**) LLE, (**c**) Isomap, and (**d**) LE.

The four nonlinear manifold learning algorithms achieved a greater than 83% classification accuracy, which was validated by five-fold cross-validation and the tuned number of nearest neighbor (*k_neighbors_*) parameter, as depicted in Figure 6. LTSA-SVM reached a classification accuracy of 93.33% with *n_dimensions_* = 8 and *k_neighbors_* = 24, LLE-SVM needed *n_dimensions_* = 6 and *k_neighbors_* = 38 to obtain optimum classification accuracy of 88.33%, LE-SVM employed *n_dimensions_* = 7 and *k_neighbors_* = 31 to achieve a classification accuracy of 83.33%. Additionally, the Isomap-SVM technique achieved the maximum classification accuracy result of 96.67% by adjusting *n_dimensions_* = 11 and *k_neighbors_* = 18, indicating significant performance improvement. Compared to the linear techniques, all the classification accuracy results are improved using the nonlinear manifold techniques. Due to the fact that the PCA technique uses only linear combinations of the original independent variables to compensate for the maximum amount of variation, only a limited amount of clustering performance can be achieved. The MDS technique is effective when the dataset is highly sparse or nonmetric, but if the original high-dimensional dataset has nonlinear relationships, as confirmed by the augmented partial residual plot, it will not be suitable [27]. Lin et al. [42] and Tsai [43] also reported that local techniques for nonlinear dimensionality reduction, such as LTSA, LLE, or LE, have two significant benefits over global approaches, such as Isomap: they accept some curvature and naturally result in a sparse eigenvalue problem. Nevertheless, neither computational sparsity nor curvature tolerance is deliberately included in the design of the local techniques. 

These characteristics appear as a result of the goal of preserving just the local geometrical configuration of the dataset. Due to the fact that they are not explicit aims but rather convenient byproducts, they are not trustworthy characteristics of the local technique. LTSA, LLE, or LE has conformal invariance that can perform unsatisfactory performance in unexpected directions, and the computational sparsity is not modifiable independently of the manifold’s topological sparsity. This study establishes Isomap as specifically designed to eliminate a well-defined form of curvature and to take advantage of the computational sparsity inherent in low-dimensional manifolds [44,45]. Both expansions are susceptible to algorithmic assessment and have been satisfactorily and successfully tested on the LIBS dataset. Overall, the obtained results demonstrate that, when nonlinear manifold learning techniques are paired with the SVM classifier model, they outperform linear manifold learning techniques.

## 4. Conclusions

This study proposed the manifold dimensionality reduction techniques and multivariate classifier model of SVM coupled with LIBS technology for classifying five kinds of aluminum alloy. The high-dimensional data and nonlinearity of the raw spectral data were confirmed by the augmented partial residual, which was represented by the polynomial fitting. The nonlinear manifold learning methods of LTSA, LLE, Isomap, and LE, and linear manifold methods of MDS and PCA were implemented as dimensionality reduction techniques and employed to retrieve distinctive variables in order to reduce the dimensions of the input dataset. The acquired significant variables were assigned as the input of the SVM classifier model for the purpose of predicting the labels of unknown aluminum alloy samples. The performance of prediction models was assessed by confusion matrix and prediction accuracy. In linear manifold learning, MDS-SVM demonstrates better results than PCA-SVM, while the Isomap-SVM shows robust satisfactory results compared to the other nonlinear manifold learning. Isomap-SVM outperforms the linear and other three nonlinear manifold learning results with a prediction accuracy of 96.67% and only 2 data were misclassified. Thereby, the investigation conducted in this study can be a superior alternative method to rapidly and accurately classify the particular sample based on LIBS and even can be used for quantitative analysis of elemental concentration.

## Figures and Tables

**Table 1:** The chemical contents in the five standard aluminum alloy samples and their concentrations.

Sample Label	Sample Code Number	Element Concentration (%)							
		Si	Fe	Cu	Mg	Mn	Zn	Ti	Cr
#1	GSB04-1991-2006	0.102	0.045	0.188	1.010	0.010	0.010	0.153	0.150
#2	GSB04-1992-2006	0.273	0.150	0.149	0.817	0.051	0.047	0.112	0.101
#3	GSB04-1993-2006	0.441	0.258	0.103	0.606	0.099	0.090	0.050	0.050
#4	GSB04-1994-2006	0.569	0.352	0.053	0.390	0.151	0.144	0.0098	0.010
#5	GSB04-1995-2006	0.751	0.459	0.016	0.219	0.207	0.201	0.0042	0.0047
